# Strengthening theories of change in women’s group interventions to improve learning

**DOI:** 10.7189/jogh.13.04098

**Published:** 2023-12-13

**Authors:** C Leigh Anderson, Rebecca Toole, Carly Schmidt, Gary L Darmstadt

**Affiliations:** 1 Daniel J. Evans School of Public Policy and Governance, University of Washington, Seattle, Washington, USA; 2 Department of Pediatrics, Stanford University School of Medicine, Stanford, California, USA

## Abstract

**Background:**

Supporting women’s groups is increasingly seen as an important intervention strategy for advancing women’s empowerment, economic outcomes, and family health in low- and middle-income countries. We argue that learning from investments in women’s group platforms is often limited by the lack of a well-articulated, evaluable theory of change (ToC) developed by those designing the programmes.

**Methods:**

We first identify a simple set of steps important to specifying a ToC that is evaluable and supports learning (what could be done). We then propose a framework in which we hope social scientists can find a common starting point (reconciling what could be and is being done). The framework emphasises identifying untested assumptions around pathways for introducing and adopting new knowledge, opportunities, technologies, interventions or implementation approaches, and pathways from group participation to behaviour change. Finally, we apply this framework to a portfolio of 46 women’s groups investments made by the Bill and Melinda Gates Foundation between 2005 and 2017 to understand the prevalence and clarity of their grantees’ theories of change (some of what is done).

**Results:**

The majority of the investment documents reviewed did not make clear the embedded assumptions or hypothesised pathways from decisions to join a group, to women's group participation, to behaviour change and and whether pathways are connected or work independently.

**Conclusions:**

We use an example from an actual investment to illustrate how this framework can support accounting for assumptions in the ToC used to guide the evaluation, the testing and measuring of mechanisms assumed to be driving behaviour change and disentangling the effects of implementationfrom context. A ToC for group-based programmes should specify in what capacities the group-based model is essential to the hypothesised pathways of impact vs. its role as an efficient delivery mechanism for programmes that would potentially generate impacts even if delivered directly to individuals. In addition, without fully specifying the motivation for individuals to change behaviour in terms of their risk/return calculus and testing underlying assumptions, we miss an opportunity to better understand the pathways for how the programme influences or fails to influence individuals’ health behaviours. However, fully specifying (and measuring) every link in the programme’s ToC is not costless. We present suggestions for developing ToCs with testable hypotheses that foster learning about why a women’s group intervention achieved or failed to achieve its intended impact.

## IMPORTANCE OF A THEORY OF CHANGE

The overarching question of this paper is: among women’s group interventions that require behaviour change for the intended outcomes, how can theories of change be strengthened to improve learning through evaluation about why an intervention has positive or negative impacts – or no impact – in order to further the evidence base? Our intent is not to critique or review evaluation theory or methods [1-3]. Rather, our point centers on an evaluator’s ability to test the logic of a theory of change (ToC) and illuminate behavioural pathways and underlying assumptions.

Attention to a well-articulated ToC guiding evaluations is not new and has been revisited since debates over evaluation methods rose to the forefront in the early 2000s and 2010s [4-6]. Nearly a decade ago, Stufflebeam and Coryn [1] performed a systematic review of 45 cases of theory-driven evaluations published from 1990 to 2009, and concluded, “if a program is effective (theory-driven evaluation), should identify which elements are essential for widespread replication. Conversely, if a program fails to achieve its intended outcomes or is ineffective, a theory-driven evaluation should be able to discover whether such breakdowns can be attributed to implementation failure (e.g. treatment integrity; Cordray and Pion, 2006), whether the context is unsuited to operate the mechanisms by which outcomes are expected to occur (Pawson and Tilley, 1997) or simply theory failure (Rogers, 2000; Suchman, 1967; Weiss, 1997b).”

Hence our goals are: 1) to revisit principles of social science research and identify key steps in specifying a ToC that is evaluable and supports learning (what could be done), 2) to propose an initial framework in which various disciplines can find a common starting point (reconciling what could be and is being done) to better specify behaviour change theories and make underlying assumptions more explicit, 3) to apply this framework to a particular but important portfolio of investments to examine their use of a ToC (some of what is being done) and 4) to illustrate use of the framework to guide improvements in using a ToC to enhance learning, based on analysis of an example from the portfolio. We draw from a 2019 portfolio evaluation of 46 Bill & Melinda Gates Foundation women’s group investments made between 2005 and 2017 [7] and use the framework to identify untested assumptions around pathways from decisions to join a group, to women’s group participation to behaviour change. We argue that women’s group evaluations are still regularly challenged to disentangle the effects of implementation from context and from theorised intervention effects, because the theory itself guiding the evaluation is incompletely specified [7]. This is a weakness at the investment level where implementers may have a clear ToC in mind but fail to translate it in a manner that provides testable hypotheses for evaluation. Thus, programmes often fail to specify an evaluable ToC that enables testable hypotheses of how – and under what assumptions – the intervention prompts the behaviour change necessary to elicit the intended outcomes and that sheds light on the intervention’s transferability across different contexts, types of women’s groups and varied implementation models.

## WHAT MAKES A THEORY A THEORY?

Two principles of the scientific method are regularities and empiricism: theorising patterns and testing subsequent hypotheses through observation and data [8]. The goal is to explain and predict changes in human behaviours – movements from the status quo in response to some intervention – rather than initial choices. But even this requires “imposing structure on individual preferences” [9]. For evaluation, tractable mathematical modeling is not required, so both the theory and empiricism can more easily accommodate considerations of the group context and deviations from a purely rational model of optimisation. Nonetheless, the patterns we posit are based on individual choice and behavioural assertions that generally hold, rather than idiosyncratic behaviours. And testing requires identification of measurable and exogenous factors. Otherwise, to attribute changes in behaviour to changes in unmeasurable phenomenon is to abandon the search for causality driving behavioural patterns and thereby interventions that will lead to systematic change.

While our application is women’s groups, we consider individuals to be the fundamental unit of behaviour change, even though individual change may be influenced by the group dynamic within which the change is made. But without individual change, groups do not change, and group behaviour is a composite of individual behavioural choices. We consider a choice to be the intersection of tastes or preferences (what one wants to do) and constraints or opportunities (what one is able to do), distinguished by the latter being measurable, and the former typically assumed to be constant over some period of analysis or, alternatively measured and taken into account analytically [10].

A well-formulated theory of behaviour change can postulate and test what perceived risks, benefits and costs matter to individuals of a particular group in a particular context. Acknowledging just what we know from individual choice theory, intended beneficiaries act based on their expected net benefits and alternatives, which vary with circumstances and demographics. But in building an evidence base for women’s groups, the “circumstances” matter. Individuals can behave differently in groups than outside groups, and in groups operating within different systems where incentives and power dynamics under formal institutions and informal norms differ along gender and other sociodemographic lines [11]. Individual choice theory for women’s group interventions must deal with the complexity of women’s preferences and constraints in low- and middle-income country (LMIC) settings, and with uncertainty, group dynamics, and interventions aimed at changing choice sets and transforming gender norms [12].

Not all theories of change are created to be evaluable; rather, they may more closely resemble logic frameworks – absent or with assumed causal pathways – or be used to help focus the intervention design, team efforts, and ongoing quality improvement. A behavioural theory goes beyond a logic model; we advance that it should make clear the set of commonly accepted or evidence-based assertions believed to hold across human behaviour in a specified context (e.g. a woman prefers more to less leisure time), that yield testable hypotheses of how behaviour will change with an intervention (e.g. increasing access to herbicide tolerant Ht maize will increase its adoption) and that make clear the assumptions (e.g. women do most of the weeding, are able to make seed choices and have sufficient income to purchase the seed) that could similarly affect a measured outcome. These are the ceteris paribus conditions: does adoption fail to occur because women are not making the seed choices, don’t have sufficient information to assess the risk/return trade-off, or don’t have access to complementary inputs? If all measurable factors driving seed choice have been articulated, measured, and accounted for – the potential counter hypotheses driving behaviour change or the lack of it – we can better understand adoption rates and the efficacy of an intervention in a particular context. If it is an unmeasurable factor, such as the “taste” for an hour of leisure that has reduced adoption, we may be stymied. But laying out all the conditions that must hold for adoption to occur, and which are assumed to be the constraints to adoption (which presumably the intervention addresses), increases the probability of learning through an evaluation about the most salient risk/return drivers of women’s choices in a particular context, and why an intervention fails or succeeds.

The hypothesised causal mechanism in our seed example is that if the risk/return calculus over seed choice can be addressed via a group intervention aimed at education and empowerment – perhaps allowing women more control over any time gained from reduced weeding hours – then the new seed will be adopted. If adoption rates are in different directions than predicted, it is helpful to be able to assume that for most women the behavioural assertion of more leisure being preferred to less holds and instead evaluate which assumptions do not hold or hold only partially [7,13]. Behaviour change that is just assumed as a result of, for example, a training or savings opportunity – without articulating the new choices or agency from consciousness raising or collective action, or financial, social, time and other personal or social costs, benefits, and risks to an individual that would lead them to adopt, participate, or otherwise avail themselves of a new opportunity or new piece of information – does not answer the questions: Why do participants move from the status quo? And under what conditions can we expect the same outcome? By laying out common behavioural assertions and the assumptions that underlie an intervention leading to a behaviour change, it is possible to test what is leading to or failing to result in the expected outcome.

In addition to informing why participants in one context move from or stay with the status quo, such theory can also help us learn how likely it is that participants in another context will do likewise. Pritchett and Sandefur [14] note the imprecision with which the salient features of a context are understood, and that “some theory is required to know what context means.” For example, while constraints to participation in a women’s savings group in one context may be low, contributing to high rates of adoption and ultimately smoothed consumption, deeply entrenched gender norms in another context may prevent the level of participation needed to achieve outcomes – and could even put women at risk of backlash after transgressing gender norms [15,16]. The absence of a theory that accounts for contextual factors weakens the likelihood of success in scaling and translating a programme to other contexts.

Limitations on translating impact evaluation results across heterogeneous country and cultural contexts (external validity) is widely recognised, but variations in implementation are also potentially critical to uptake and impact [14,17]. Pritchett and Sandefur [14] discuss that “in the course of design and implementation, programs have to make choices from a high-dimensional space of design attributes”, and that a programme – microcredit, savings, conditional cash transfer – “is just an instance of a class” [14]. How a programme is delivered and messaged – who will be eligible, at what price, for what duration, and with what conditionalities – can directly affect the time, social, and financial costs to a group member, and her perception of the risks and potential benefits of participating. In women’s groups, implementation details such as membership criteria, external networks, and group governance can be crucial to understanding whether a woman chooses to join a group, is able to envision broader possibilities, and actively saves and participates [13,18,19]. Without a ToC, it is difficult to know pre-evaluation what the most important margins are to vary and measure, and post-evaluation what the likely programme results will be when contexts and implementation change.

Fully specifying the theory is, arguably, particularly important for evidence on women’s group interventions in LMICs. The most generalisable theories of change evolving from individual choice theory have been largely modeled by and tested on dominant market actors, i.e. men and western university students [20]. But we know, for example, that many experiments find behaviours anomalous to, or not encompassed by, that predicted by traditional rational choice theory: for example, fairness matters, losses hurt more than commensurate sized gains help, and choices can be inconsistent across menus [21,22]. When assumptions of perfect information are relaxed, decision-making under uncertainty leads to biases including for the status quo and that individuals will choose lower potential returns with known probabilities over higher potential returns with unknown probabilities [4,5,23]. Individuals act on risk perceptions influenced by a suite of qualitative dimensions which may well differ from measured risk and those of the funder or implementer. Evidence from experiments suggests, for example, that individuals regularly overestimate the risk of small, dreaded, unfamiliar, unfair, and involuntary events, and that individuals are loss averse and more risk seeking over losses than gains [24-28]. These examples often surface socio-economic patterns, but there are also gendered patterns in social preferences; some findings suggest, for example, that women are more generous with those whom they know and less with strangers, and more positional over relative well-being but less so over absolute, than are men [29,30]. Finally, empowerment itself is about agency and changing the choice sets women envision [31] – through consciousness-raising processes, social learning and networking – and since we know that preferences are not stable over time and can be menu-driven [32], when the menu itself changes due to the intervention, this presents an empirical challenge.

Building out non-mathematical theories of women’s group participation and outcomes allows for less structure and can accommodate theories where individual, unobservable preferences change. Nonetheless, if multiple observable and unobservable mechanisms are presumed to feed into each other and no node in a system is exogenous or fixed (that is, if no causal pathway is specified because everything is said to affect everything else and no basic behavioural assertions are assumed to be held in common), then at least over some time frame specifying testable hypotheses is a challenge, and therefore learning from supporting or refuting those hypotheses is limited. Benefits, costs and risk evolve from systems, group dynamics, norms and learning, but since their relative value is revealed at a point in time through individual choice – which we do assume exists at the margin or arises through increased agency, however constrained, for example by social and gender norms – we use a simple but powerful theory of behaviour change as illustrative. We argue that for an individual to voluntarily move from the status quo, the benefits they expect (given uncertainty) must outweigh the costs – financial, time, social, psychological, physical, etc. – however they weigh and experience them and for whatever choices they perceive.

## METHODS

### Theory of change applied to a women’s group portfolio of investments

Our primary objective is to illustrate the importance of strengthening theories of change in women’s group interventions to support evaluation with learning. We seek to clarify the role of theory in improving the ability to contribute to broader sectoral learning. We define theory as well-established behavioural assertions (e.g. an individual seeks to improve her well-being) and the assumptions as to what will move an individual from their status quo, supported by a strong body of evidence that yields testable hypotheses (e.g. higher perceived net benefits, or reduced risk). The portfolio evaluation of Bill & Melinda Gates Foundation-funded women’s group interventions [7] serves as a case study to explore this concept in practice.

From 2005 through 2017, an estimated 330 million US dollars ($) was invested by the Bill & Melinda Gates Foundation in programming and research that to one degree or another involved women’s groups. These 46 grants, 42 of which involved a group-based intervention and four of which funded research or technical assistance, were designed with a number of goals in mind and many were considered successful. Yet ultimately our ability to learn about the impact of women’s groups on economic and/or health outcomes or empowerment from these investments is limited, in many cases because of an absent or incomplete ToC. There is often ample evidence from previous interventions and relevant social science research to support and make assumptions explicit, and well-thought-out behavioural pathways may have been in the minds of grantees, but this was seldom reflected in a documented ToC – leaving it open to multiple interpretations.

All of the 42 interventions relied upon individuals moving from the status quo to achieve intended outcomes. Of the 42 grants, just over half (n = 24) had an explicit ToC, meaning a ToC was clearly described in the proposal, and 20 included some discussion of an individual moving from the status quo as a result of the intervention. Approximately ten of the grants that did not specify behaviour change in their ToC were focused on capacity building of implementing organisations or systems change. No grants clearly included an individual’s decision to join (or leave) a group in their ToC – we assume this reflects the focus of most grants on interventions for existing groups, rather than creating new groups.

Of the 42 grants with an intervention, all were motivated by assumed benefits, while only 26 discussed costs or risks to individuals of participating in the intervention. Only two grants clearly incorporated risks or costs to individuals of participation into their ToC. In sum, a minority of investments specified a ToC that would enable a high-quality evaluation with learning – without additional information or input from the designers of the intervention.

### What makes a group a group?

We recognise that interventions in women’s groups may be particularly challenging to model and test because of group dynamics, collective action considerations, and context-dependent preferences. Ostrom (2009) [33] and others long ago noted “how a theory of boundedly rational, norm-based human behaviour is a better foundation for explaining collective action than a model of maximizing material payoffs to self.” The difficulty of testing such theory of the group context has also been noted, considering trust and reciprocity in which members “assess the actions of others” to inform their own decisions, and because a component of many women’s groups is to expand members’ agency, thereby enabling changes in attitudes, preferences, social interactions, and systems [34]. Over the last 20 years, a number of important theoretical and empirical advancements have helped to uncover the key motivators of collective action. The social identity model of collective action (SIMCA) [35] synthesised three distinct, albeit interconnected, processes that facilitate collective action. According to SIMCA, people will engage in collective action to advance the interests of a group to which they are committed (social identification [36]); when they appraise that their group is unjustly deprived relative to other groups (injustice [37,38]); and believe that, by acting, they can effectively change the existing state of affairs (group efficacy [39]). Feminist economic theory furthermore recognises individual-level human capital factors, meso-level normative factors and structural factors in governing behaviour [40]. Rather than assuming that unobservable factors are constant and focusing on more measurable constraints and opportunities (time, access, finance) that change with an intervention, consideration must therefore also be given to the possibility that participation changes a member’s preferences or view of feasible choice sets. For example, instead of attributing a woman taking on more credit risk as only being driven by a group insurance mechanism, it could be attributed to increased risk tolerance or changing aspirations, perhaps aided by increased agency and a change in power dynamics within the household or community.

**Figure 1:**
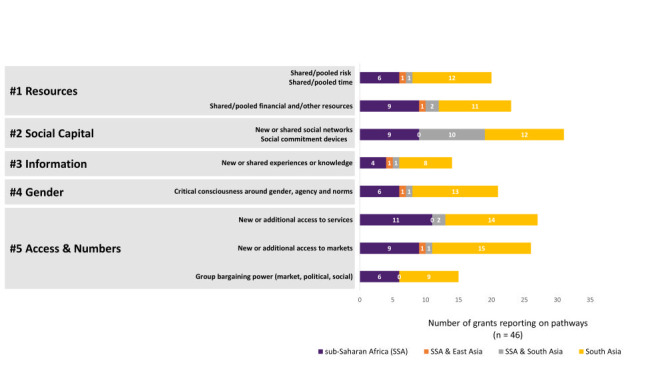
Pathways through which group-based interventions can lead to benefits. Grants can have more than one pathway. Some grants operate both in sub-Saharan Africa and South Asia.

Pathways that make groups different from individuals involve how groups rearrange or add to resources to generate economic, health, and/or empowerment benefits. In **Figure 1**, we identify pathways that represent key avenues through which groups can provide benefits to members that are not necessarily individually attainable. We identified these pathways based on review of documentation of the 46 women’s group grants.

### Building an organising framework

**Figure 2:**
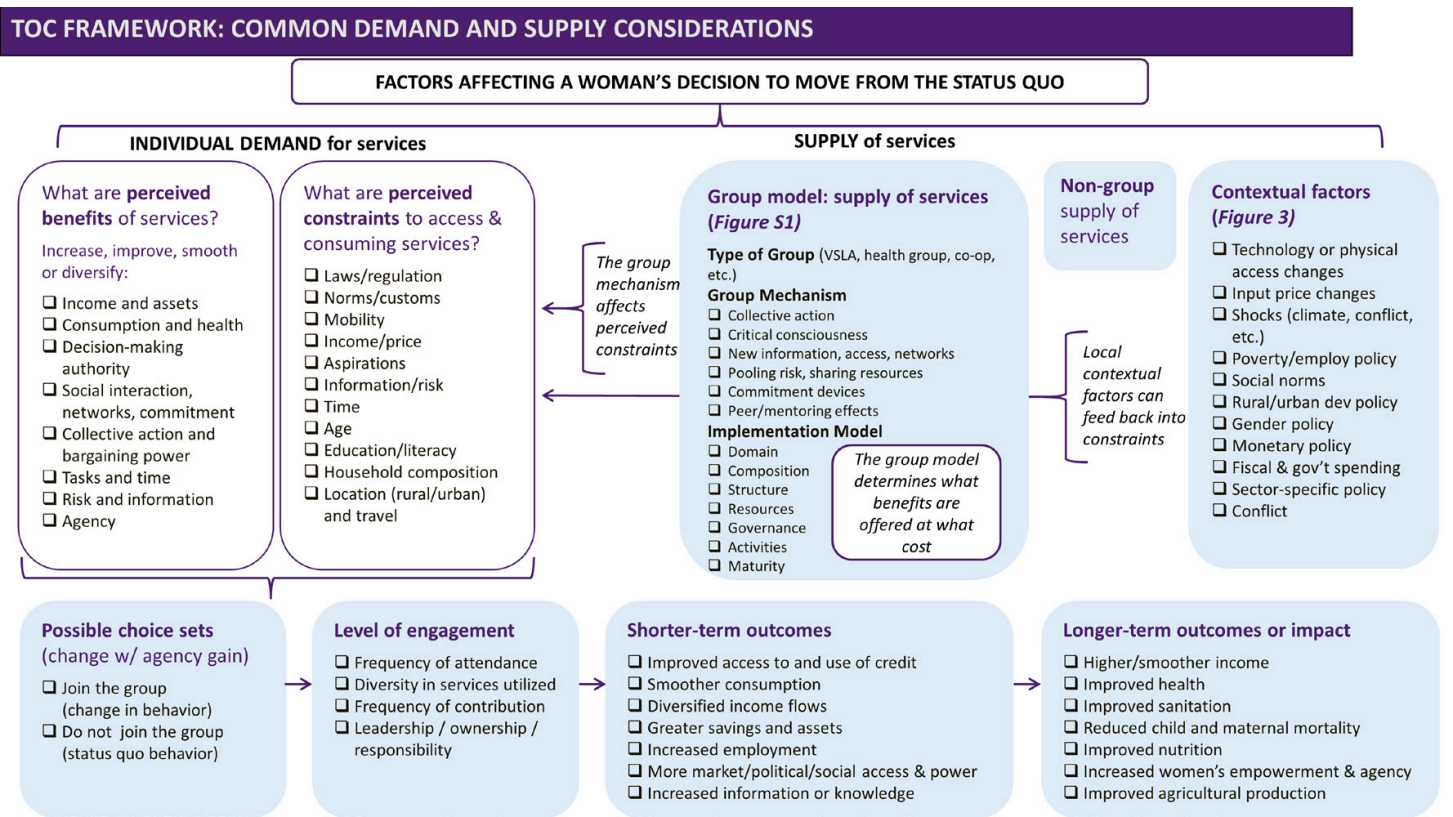
Theory of change (ToC) framework illustrating key factors and assumptions underlying pathways to outcomes unique to group participation.

**Figure 3:**
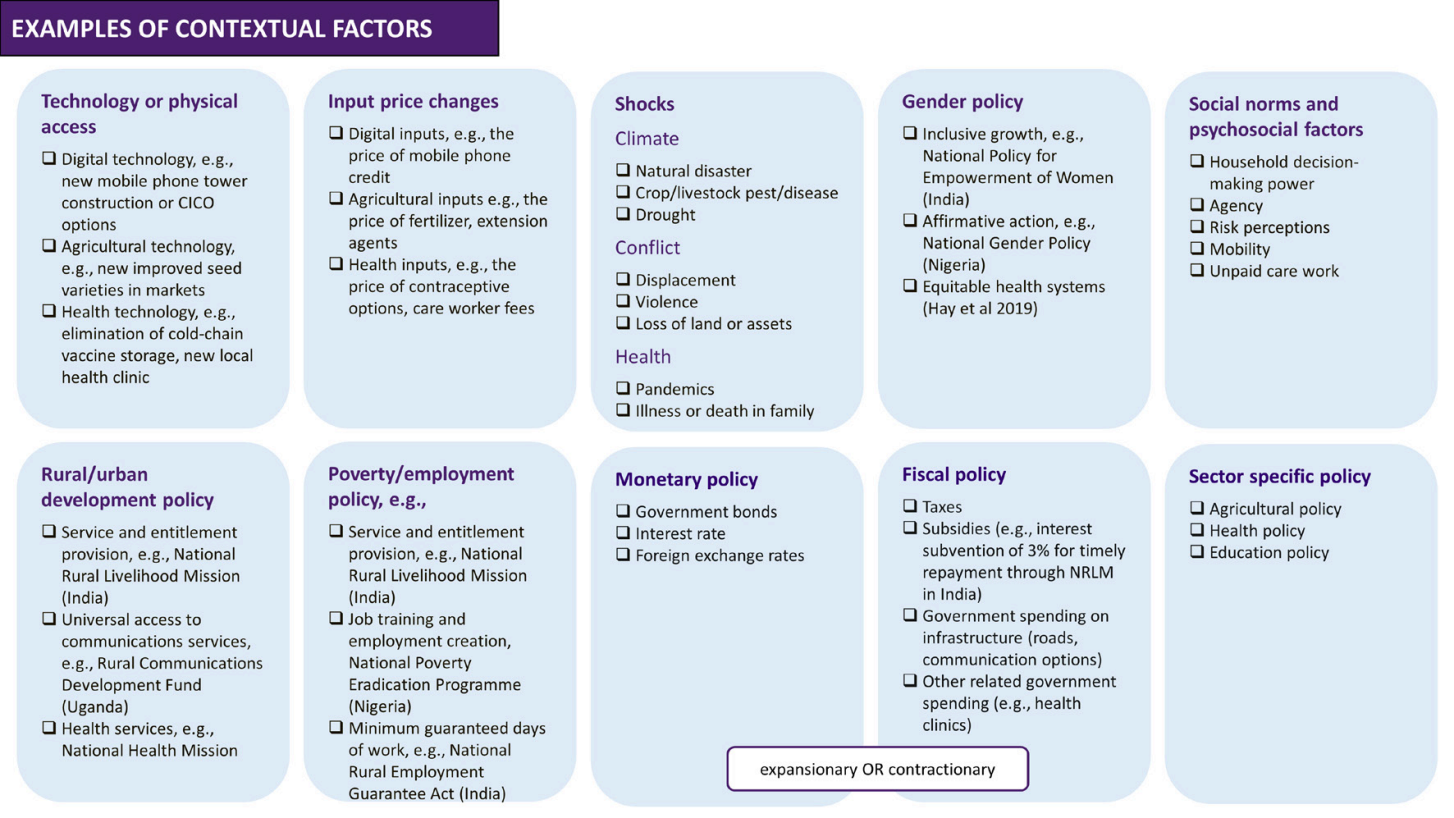
Contextual factors underlying pathways to behaviour change through group participation.

Though obviously not exhaustive and not meant to be prescriptive, the purpose of a framework (**Figures 1**, **Figure 2** and **Figure 3****,** Figure S1 in the **Online Supplementary Document**) is to begin to articulate common hypothesised factors that could affect a woman’s decision to engage with a group and the nature of that engagement [41].

We envision three uses for the framework. The framework intends to prompt implementers to more precisely specify how the intervention changes perceived costs or benefits and thereby behaviour change through a pathway unique to a group (**Figure 1**) and to highlight assumptions in their ToC about co-variates that may be responsible for behaviour change in addition to the particular intervention being evaluated. Thus, the framework may help implementers articulate testable hypotheses. The framework may further remind evaluators to account for co-variates, including the policy and market environment where feasible, and to document the particular pathways unique to group implementation mechanisms. Thus, the framework may help with designing evaluations. Finally, we envision this as a “living” framework that others will build on as we learn of the most salient demand, supply, and contextual factors to consider in terms of variation in, and generalisability of, women’s group outcomes.

**Figure 1** theorises “group-based” pathways. But a complete ToC would a) specify what motivates intended beneficiaries to change behaviour in terms of their expected benefit-cost calculus; b) account for underlying assumptions including contextual and implementation heterogeneity, group dynamics and system interactions, and c) put forward testable hypotheses. For group-based interventions, a complete theory of why a woman may choose to participate or not, and why an intervention may or may not lead to behaviour change, requires that the costs of group engagement and the cost of moving from the status quo also be articulated. The framework therefore posits a set of theory-based factors or variables that can affect behaviour change and women’s group individual-, household-, group-, and community-level outcomes, aimed at highlighting what is specific to group-based interventions. The choice of factors to measure depends on the context, the group, the intervention, and specific evaluation questions – factors and questions that should be drawn out of the ToC.

The second component of the framework considers women’s individual decision-making and demand for services, given the availability and supply of those services through groups (**Figure 2**). We list some of the most commonly mentioned perceived benefits and constraints to both joining and engaging with a women’s group. We distinguish perceived from actual as being relevant to behaviour, and note that given uncertainty, these are expected benefits and costs. We consider most women to prefer more of these benefits (time, income, assets) to less of them, acknowledging important work on biases in decision-making under uncertainty and how poverty itself can affect aspirations [42]. A consideration of perceived net benefits will influence a women’s choices (to join or not, when and how much to engage with the group, and in which aspects).

The third component of the framework covers the possible shocks, structural interventions, or market or policy changes that affect behaviours (**Figure 3**). Just as it is important to clearly lay out the “all else equal” assumptions that underlie behaviour change, a ToC should consider changes in the enabling environment that affect women’s constraints in engaging as a group member, or the supply of group services. Considering these is also important to interpretations around the external validity of the intervention.

An additional component of the framework (Figure S1 in the **Online Supplementary Document**) details variations in the group model, and how the intervention is delivered. We recognise that how different services are supplied matters to access, alongside the constraints which include limited agency. Leadership, trust, and the group culture will likely affect a woman’s choice, if she has one, between receiving a service privately or within a group. Documenting these implementation differences is important to construct validity.

## RESULTS

### Applying the framework

In this section, we present an example from a portfolio of Bill & Melinda Gates Foundation investments in women’s groups to illustrate how laying out a complete ToC at the outset can help to inform an evaluation. In selecting this evaluation, we were agnostic as to the logic of the ToC; rather, we sought to illustrate how the ToC informed, or fell short of guiding evaluative processes that led to answering the research questions and informed learning about why group-based behaviour change did or did not occur as a result of the intervention. The selected example met the following criteria, as coded during the portfolio review: (i) the evaluation included a ToC, (ii) the evaluation explicitly stated the ToC, (iii) the specified ToC considered individual behaviour and (iv) the ToC addressed how individuals might change their behaviour in response to the group-based intervention. Of the investments reviewed, four investments met these criteria. For illustration, we use the Parivartan programme to draw out what other questions might have been answered with a broader-based framework such as that illustrated in **Figure 1**, **Figure 2** and **Figure 3** (which was not available during design). One dimension of individual behaviour that is not explicitly addressed within the ToC in this example is the benefit-cost calculus of joining and participating in a group – that is, the financial and time costs, and potential risks to personal autonomy, indebtedness, social sanctioning, etc. Our comments are based on what we were able to publicly access, and we acknowledge that more details may be available to fill highlighted gaps. As is often the case for published papers, any results from a process evaluation – if conducted – are not reported, so we have limited information on programme implementation. Finally, we are not assessing the value of the programme, or the design, quality or ability to establish causality; rather, our focus is more narrowly on how a more detailed ToC with testable hypotheses could increase learning.

The Parivartan programme endeavored to increase demand and also the practice of family health and water and sanitation behaviours [43-46]. It formed and/or nurtured approximately 19 000 health-focused self-help groups (SHGs) in Bihar, India with women who belonged to marginalised communities, such as scheduled tribes or castes, as well as Muslim women. Groups were comprised of women of, and beyond, reproductive age. Parivartan incorporated eight participatory sessions into weekly SHG meetings to improve reproductive, maternal, newborn and child health and nutrition (RMNCHN). It also promoted collectivisation – developing the collective efficacy, agency, and action of the group – with support from community health facilitators (*Sahelis*). More specifically, the programme purported that “community mobilisation (collectivisation) aimed not only to empower key populations as a group for vulnerability reduction, but also to enhance their self-efficacy (defined as the ability to make decisions about one’s own behaviours) which in turn influences the adoption and maintenance of healthy behaviours.” The standard (comparator) SHG programming focused on financial literacy and savings support and services, combined with unstructured health and social messages.

A hypothesis underlying the group-based delivery of the targeted health sessions was that collectivisation spills over to the individual. Parivartan’s ToC specified that developing collective efficacy of the group would in turn improve the self-efficacy and self-confidence of individual members, allowing for individuals and hence groups to advocate for services from local administrative agencies and to negotiate within the household to counter dynamics that impeded health-promoting behaviours. The ToC maps to pathways for Social Capital, Information, Gender, and Access and Numbers in **Figure 1****.**

Supply-side interventions to strengthen the enabling environment were implemented alongside Parivartan, as individual behaviour change was deemed necessary but not sufficient for systems change. Frontline workers received training and job aids to implement critical RMNCHN interventions, and facilities received support to improve basic emergency obstetric care services [43-45,47].

Saggurti et al.’s [45] impact evaluation of Parivartan used a difference-in-difference design to compare health and empowerment outcomes between married women aged 18-49 years who had a child under one-year of age and belonged to SHGs that incorporated the weekly health-focused sessions (i.e. health-layered SHGs) with women who joined standard SHGs (the comparison group). The research team collected data from both group leaders and members on 1) sociodemographic characteristics, such as age, parity, occupation, literacy, and caste, 2) group membership, 3) the efficacy, agency, action and cohesion around maternal and child health, and 4) health outcomes.

In line with the central hypothesis of the ToC, the evaluation emphasised learning about changes in behaviour which are theorised to be spurred by collectivisation. However, there were several limitations within the ToC that were also reflected in the evaluation. The intensity of group processes, including the level of participation, quality of facilitation processes, and quality of group dynamics were not well defined, and thus, ways in which group participation led to collectivisation and changes in health-related behaviours were unclear, especially considering that many women in the groups were not of reproductive age. If a process evaluation was conducted, it is not publicly available so we cannot assess several components of **Figure 2** – in particular, the level of engagement and the profile of women who engaged with the SHG. The evaluation does not discuss whether the sample selected is typical of group members.

Second, the ToC did not directly address assumptions underlying the availability or affordability of health services, the capacity of the health work force or the quality of delivery (all of which could affect women’s ability to secure RMNCHN care). In addition, the impacts of efforts to strengthen the quality of the health system were not measured within the evaluation. Similarly, the ToC did not specify assumptions for external market factors that could constrain behaviour, such as food prices and security (which could affect women’s health and ability to introduce complementary feeding). In sum, many of the contextual factors outlined in **Figure 3**, if noted, were not discussed. With these limitations, the ToC did not fully specify a theory of behaviour change, as it was limited in its identification of health system- or market-related factors that influenced whether an individual took up the promoted health behaviours.

**Figure 4:**
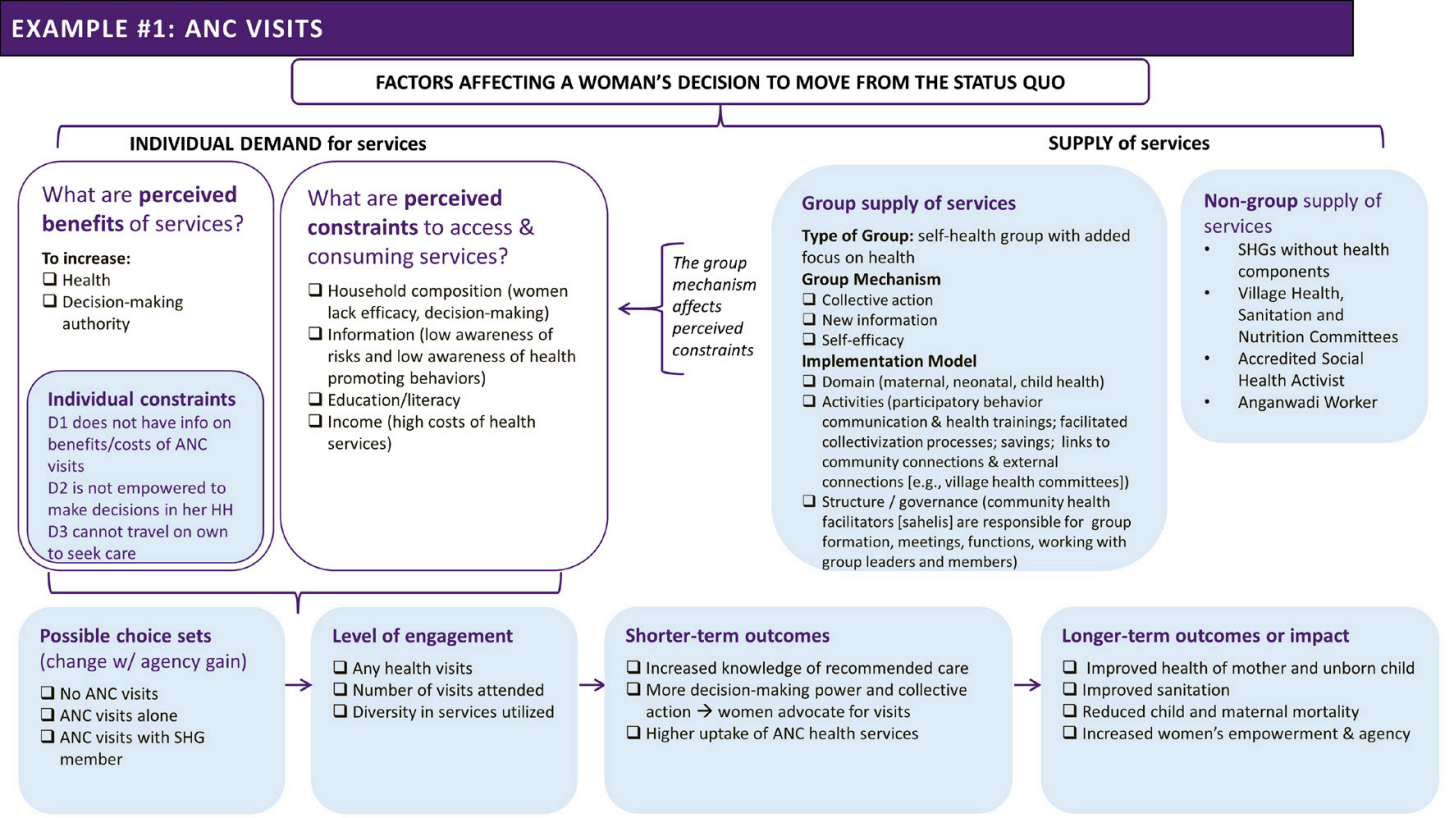
Theory of change (ToC) for increasing antenatal care (ANC) visits through the Parivartan programme, developed through applying the framework outlined in **Figure 2****.**

**Figure 5:**
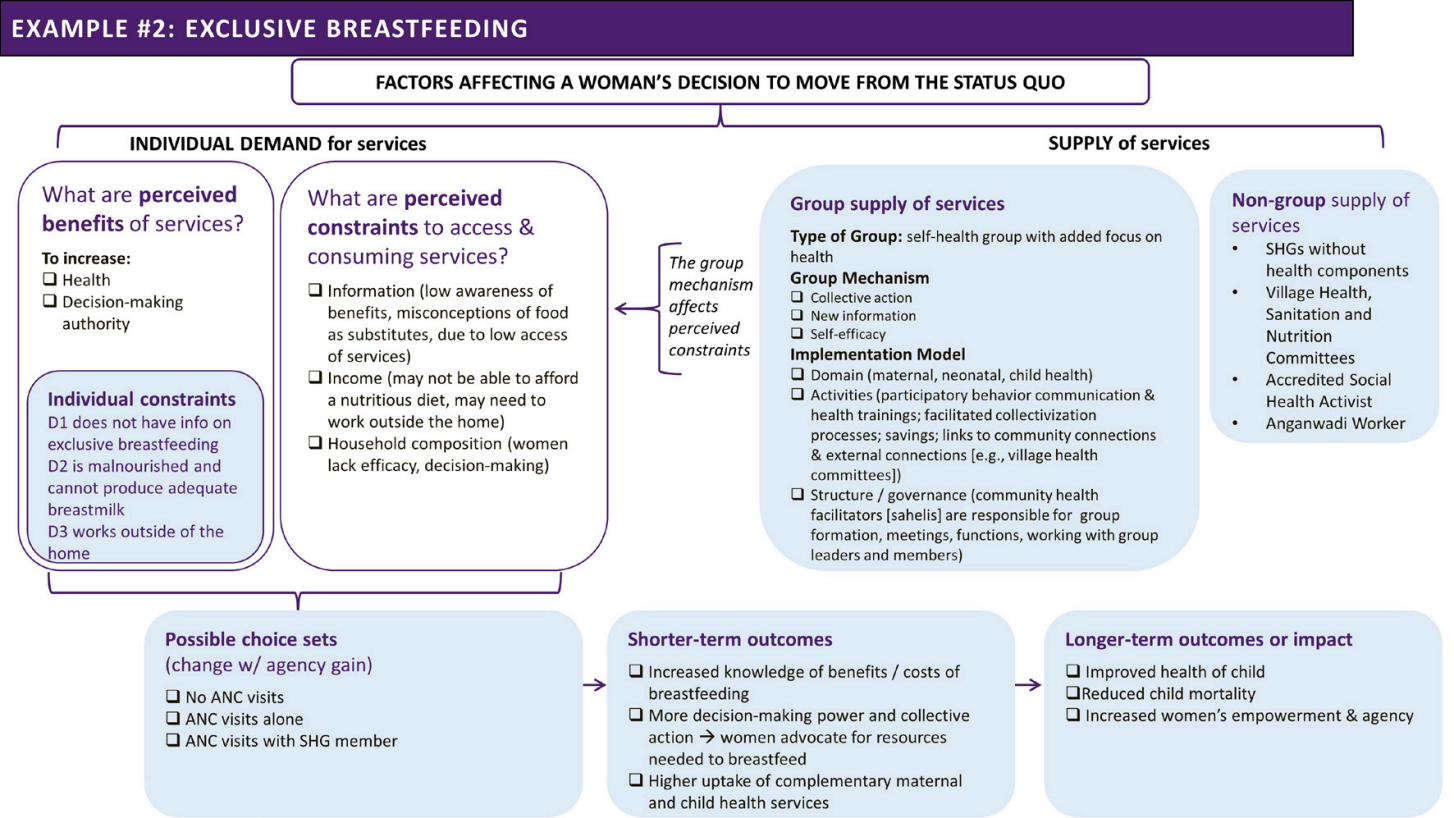
Theory of change (ToC) for increasing exclusive breastfeeding through the Parivartan programme, developed through applying the framework outlined in **Figure 2**.

In the sections below, we build on the ToC of the programme and apply our framework to specify a potential path to individual behaviour change tested with respect to attending four or more antenatal care (ANC) visits (**Figure 4**) and exclusive breastfeeding (**Figure 5**), two health behaviours promoted by the sessions.

#### ANC visits

In theory, women in Bihar could access antenatal advice and care at local facilities. In addition, Auxiliary Nurse Midwives, Accredited Social Health Activists, and Anganwadi Workers were cadres of frontline workers present in local communities to provide health services and link individuals to care. However, under the status quo at the outset of programme implementation, only 10 percent of women attended four or more ANC visits as recommended, presumably due to both supply-side and demand-side challenges. On the supply side, women may experience embedded systemic gender-based biases and intersectional discrimination against marginalised sub-populations, and service providers may provide low quality or insufficient care [11,48]. On the demand side, women may lack information on the value of these visits as well as decision-making power in determining when and how intensively to access care and may face constrained mobility. Low income and educational attainment may also dampen demand for services.

By joining and attending the group meetings facilitated by the programme, the ToC hypothesises that women will receive information about the value of ANC visits and that the process of collectivisation will empower women to negotiate for access to ANC services. Under this assumption and the assumption that supply-side constraints are not present, women can then choose to attend ANC visits, alone or with a fellow SHG member or family member, or abstain from visits. Following this choice, women can decide when to initiate ANC visits, how many ANC visits to attend, and the diversity or intensity of health services accessed during these visits. In the short term, these expanded choices can increase knowledge of recommended care, decision-making power over health decisions, adoption of recommended practices, and uptake of antenatal health services. Hypothesised longer-term impacts include improved health of the mother and newborn infant, improved sanitation, and reduced newborn and maternal mortality and morbidity.

The evaluation found no changes in the share of women who attended four or more ANC visits relative to the control group. However, women were more likely to report that a group member accompanied them to one or more ANC visits, suggesting that the layering of health messages onto SHGs encouraged take-up of health behaviours to some degree, but was not sufficient to advance the health goal of attending four or more ANC visits. As designed and with the information available, we cannot conclude why collectivisation was insufficient. That is, we cannot determine whether the persistent barriers women faced in securing ANC visits reflected implementation failures (e.g. ANC sessions were not conducted; women in the sample did not attend the sessions on ANC; or sessions were ineffective in conveying the importance of ANC visits), demand constraints (examples are suggested in **Figure 2**, e.g. women lacked the autonomy to allocate time to make these visits or lacked affordable and accessible transportation) or supply-side constraints stemming from the health system (examples are suggested in **Figure 2**, e.g. service providers did not make supplies and services accessible, they continued to charge undue fees or discriminate in providing care, for example in not providing respectful care). With the information available we also cannot rule out other contextual factors, as presented in **Figure 3**. Alternatively, women may simply have not wanted to attend ANC visits. An implicit assumption of the ToC is that ANC visits offer net positive benefits (**Figure 2**) for expecting women. We lack the data needed to validate the assumed benefits – that is, the care provided during these visits and women’s perceptions of the value of visits. However, if the assumption that these visits provided value was incorrect, then we would not expect women to attend more ANC visits, even if Parivartan achieved its goal of collectivisation and women did not face demand constraints.

#### Exclusive breastfeeding

Only a minority of women exclusively breastfed under the status quo (33 percent at baseline in the control group). Women may not have information on the benefits of exclusive breastfeeding, or they may hold misconceptions that introducing other liquids or foods provides equivalent benefits or that it is necessary to augment breastfeeding. Perhaps more importantly, women may not have adequate information about the dangers of not exclusively breastfeeding (contaminated water or feeding utensils) and the role that introducing other foods/liquids have on reducing a mother’s ability to produce a sufficient supply of breastmilk. Breastfeeding requires learning and troubleshooting to overcome challenges, as well as adequate privacy and time. Working outside the home and the demands of caregiving to other children or other forms of unpaid care work inside and outside the household may also pose challenges.

By joining and attending the group meetings facilitated by the programme, the ToC hypothesised that women would gain knowledge about exclusive breastfeeding and its benefits and that the process of collectivisation would support women in learning how to breastfeed or empower women to negotiate for the support needed to breastfeed. Under this assumption, women could then choose exclusive breastfeeding, partial breastfeeding (supplementing breastmilk with additional foods or liquids), or not breastfeeding. Following on this choice, women could then determine how long to exclusively breastfeeding. Over time, expected impacts included improved survival, health and cognitive development of the child.

The evaluation found a larger increase in exclusive breastfeeding among the beneficiaries of the health messaging in the intervention group than in the comparison group, indicating that integrating health sessions into SHG activities encouraged women to take up this health behaviour. But, as designed and with the information available, we cannot determine which constraints the programme relaxed or which facilitating factors were most effective under what circumstances. Visits by fellow SHG members in the two days after birth increased relative to the comparison group, which may have helped mothers learn and practice breastfeeding and address breastfeeding problems. However, other potential pathways might have included improved knowledge from the health sessions alone, or policy change (**Figure 3**) or boosted women’s negotiating power to advocate for the time, resources, and support needed to exclusively breastfeed (**Figure 2**). We are also unable to determine how other confounding variables that determine the ease or cost of substituting for exclusive breastfeeding – such as the availability and cost of other sources of nutrition, the mother’s control over her own time use, etc. – may have affected exclusive breastfeeding rates.

### Limits to learning with the evaluation

Overall, the results of this evaluation revealed improvements in the majority (e.g. exclusive breastfeeding), though not all (e.g. four or more ANC visits), of the targeted health behaviours [43,44]. But without fully specifying the motivation for individuals to change behaviour in terms of their risk/return calculus and testing underlying assumptions, we miss an opportunity to better understand the pathways for how the programme influences or fails to influence individuals’ health behaviours. Without testing assumptions about the enabling environment for behaviour change, we fail to learn from null results because we cannot rule out other factors.

However, fully specifying (and measuring) every link in the programme’s ToC is not costless. Some outcomes – in this example and in general – could arise from a number of unaccounted for factors, while for other outcomes, fewer counter hypotheses might exist. In the case of Parivartan, attending ANC visits, for example, depends on a number of supply factors as well as the behaviours and beliefs of women and others in their household and community. Exclusive breastfeeding depends less on supply factors and more strongly on whether women successfully learn how to practice this behaviour, whether their household or work dynamics enable them to take the time needed to provide it, and whether women desire to breastfeed.

Ruling out the demand-side hypotheses underlying an impact (e.g. that the programme increased women’s perceptions of the benefits of exclusive breastfeeding or helped women learn how to breastfeed through the information sessions and support from SHG members) or a lack of impact (e.g. that women did not attend four or more ANC visits because they could not obtain permission to travel) would have required additional data to be collected through the household survey. The relevant questions may have been excluded to minimise the time burden of respondents or to reduce field costs. The costs of ruling out the supply-side hypotheses are likely even higher, requiring additional data collected from health workers and potentially administrative data from the health system. Yet, without testing the programme’s assumptions on the role of various demand- or supply-side barriers, it is challenging to draw on the evaluation results to determine which and how different components of the programme could be strengthened and to assess programme generalisability. Given restrictions on resources and time for surveys, systematically mapping out potential pathways and co-variates may allow for prioritisation of measures most likely to maximise learning and, in this case, health impact.

## DISCUSSION

A complete ToC clarifies if and how programme components offer different hypothetical pathways to outcomes and impacts and identifies any potential unintended consequences [12,16,49]. The many investments within the portfolio we feature involve multiple pathways – each of which could affect the risk, benefit and cost of behaviour change differently. The theories of change specified in the investment documents do not make clear the embedded assumptions or hypotheses around whether each pathway works independently or if connected, how. Clarity within the ToC around how components might hypothetically interact would aid evaluators in identifying and understanding the central hypothesis for a bundled programme.

Lastly, a complete ToC for group-based programmes would specify in what capacities the group-based model is essential to the hypothesised pathways vs. its role as an efficient delivery mechanism for programmes that would potentially generate impacts even if delivered directly to individuals. This may differ by programme component, e.g. information may offer equal potential impact regardless of whether it is delivered to an individual vs. a group, but building collective cohesion and efficacy to enable members to negotiate for improved services may only be achievable if women are engaged as a group. Specifying this distinction goes along with the importance of acknowledging and controlling for implicit assumptions.

Our approach is intended to be illustrative, but we nonetheless acknowledge the limitations of an initial sample of 46 investments from the same donor and our selection of one investment as representing the state of theories of change for women’s groups. We also note that our information on these programmes is limited to the grant documents and publicly available research papers. These resources often lack information on programme implementation – whether SHGs effectively recruited target women as members or functioned as intended. We acknowledge that our analysis provides but a glimpse into the programming, but it represents the glimpse available to the broader community from which to learn.

We also acknowledge that implementers may conceive of their programme logic and casual linkages differently depending on the decisions at hand. For instance, a logic model may be used in diagnosing the fidelity of implementation, or a simplified ToC may be used to appeal to donors. To be evaluable, however, we argue that a ToC must include testable hypotheses of how behaviour will change and identify the other supply and demand factors that could lead to a measured outcome if the assumptions do not hold.

## CONCLUSIONS

Even if good evaluation techniques can estimate impact, the ability to foster learning about why and how an intervention achieved or failed to achieve its intended impact is compromised without specifying testable hypotheses from a ToC and without ensuring that both the impact evaluation and any accompanying process evaluation align with the ToC. An under-specified ToC perpetuates evaluation with limited learning by constraining the ability of decision-makers to harvest and synthesise learnings, impeding the advancement of the knowledge base more broadly, and, ultimately, the sector’s ability to effect change at a broader scale due to limitations in the generalisability of the knowledge.

## Additional material


Online Supplementary Document
