# Supplementary material for: Strengthening theories of change in women’s group interventions to improve learning
Source: J Glob Health. 2023 Dec 13;13:04098. doi: 10.7189/jogh.13.04098 (PMC10716675; doi:10.7189/jogh.13.04098)
Supplement: Online Supplementary Document [file jogh-13-04098-s001.pdf]

Supplementary file

Strengthening Theories of Change in Women's Group Interventions to Improve Learning

Figure S1. Implementation considerations specific to programs delivered through women's groups

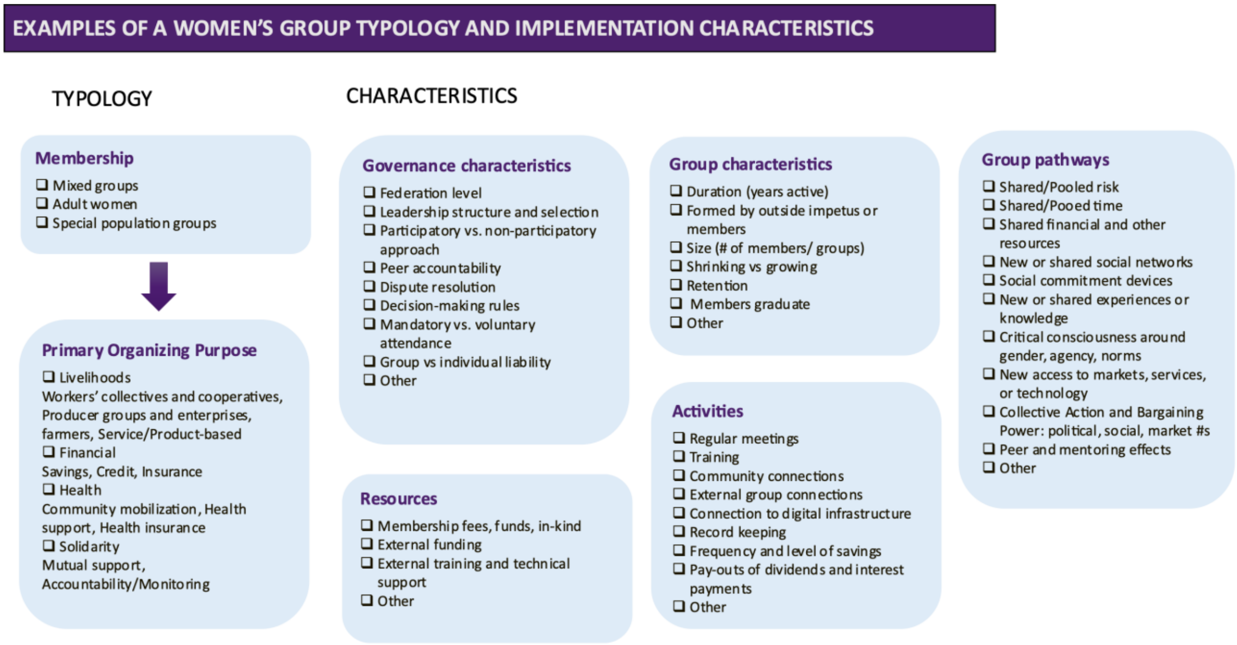

Implementation considerations

Women's groups come in many forms, with potentially important differences in perceived constraints and pathways for delivering impact. These differences are outlined according to how and what groups provide, and with whom. For example, the frequency and location of group meetings and any membership fees or saving requirements will affect the time and financial costs to members, and the network of other organizations the group is associated with may affect the

number of members who can be pooled across which risk factors, and thus its collective action potential. For a thorough discussion of group types, please see [49].
